# A common definition should be used in future studies of NSC

**DOI:** 10.1186/s13049-021-00851-z

**Published:** 2021-02-16

**Authors:** Kirsi Kemp, Reija Mertanen, Leila Niemi-Murola, Lasse Lehtonen, Maaret Castrén

**Affiliations:** 1 Department of Emergency Medicine and Services, Helsinki University Hospital, and Emergency Medicine, Helsinki University, Helsinki, Finland; 2 Department of Anesthesiology and Intensive Care Medicine, University of Helsinki and Helsinki University Hospital, Helsinki, Finland; 3 Department of Public Health, University of Helsinki and Helsinki University Hospital, Helsinki, Finland

Dear Editor,

We have read with great interest a recent commentary, “How should nonspecific complaints be defined? Comment to: “nonspecific complaints (NSCs) in the emergency department” [1] concerning our systematic review [2].

In the commentary, our decision not to include studies by the BANC group was questioned. As the BANC group has laid a solid groundwork for the study of NSC, we have cited the BANC group studies throughout our review, starting with the definition of NSC. As the concept as defined by Nemec et al. is relatively new [3] and many authors have used different names for the condition (for example Djärv et al. [4]), we extended the search terms and included studies beyond this definition. Indeed, the BANC group itself suggests that the presentation of generalized weakness should be subsumed to NSC [5].

The BANC group is referring to their original definition of NSC excluding patients in triage categories 1,4 and 5. Triage for the older adults is challenging, and especially challenging when there is a lack of a specific complaint [6]. In our opinion, if a patient without a specific complaint was occasionally triaged into these excluded categories, they would still be presenting with an NSC.

In our article, we have stated the exclusion criterion of many studies from the BANC group being the patient selection in only two out of five triage categories. However, this was merely the shared attribute in the BANC group studies, and we regret having expressed the exclusion criteria poorly. Detailed reasons for exclusion of potentially relevant studies can be inspected in Appendices 2 and 3 of our review.

Not many studies have been published on NSC, and even those are heterogenous. This is reflected in our review, which is the first of its kind. We wholeheartedly agree with the BANC group, that studies on this topic should be based on a common definition to improve the quality of future reviews.

## Data Availability

N/A
